# Sgh1, an SR-like Protein, Is Involved in Fungal Development, Plant Infection, and Pre-mRNA Processing in *Fusarium graminearum*

**DOI:** 10.3390/jof8101056

**Published:** 2022-10-08

**Authors:** Guanghui Wang, Peng Sun, Zhongjuan Sun, Jindong Zhu, Dan Yu, Zhe Tang, Zonghua Wang, Chenfang Wang, Huawei Zheng

**Affiliations:** 1 College of Plant Protection, Northwest A&F University, Xianyang 712100, China; 2 Fujian Key Laboratory on Conservation and Sustainable Utilization of Marine Biodiversity, Fuzhou Institute of Oceanography, Minjiang University, Fuzhou 350108, China; 3 College of Plant Protection, Fujian Agriculture and Forestry University, Fuzhou 350002, China; 4 State Key Laboratory of Agricultural Microbiology, Hubei Hongshan Laboratory, The Provincial Key Laboratory of Plant Pathology of Hubei Province, Huazhong Agricultural University, Wuhan 430070, China; 5 College of Forestry, Northwest A&F University, Xianyang 712100, China

**Keywords:** *Fusarium graminearum*, wheat scab, SR protein, Gbp2/Hrb1 ortholog, RNA processing

## Abstract

Serine/arginine (SR) proteins are essential pre-mRNA splicing factors in eukaryotic organisms. Our previous studies have shownthat the unique SR-specific protein kinase Srk1 is important for RNA splicing and gene transcription in *Fusarium graminearum*, and interacts with two SR proteins, FgSrp1 and FgSrp2. In this study, we have identified an SR-like protein called Sgh1 in *F. graminearum*, which is orthologous to budding yeast paralogous Gbp2 and Hrb1. Our data have shownthat the Sgh1 is involved in vegetative growth, conidiation, sexual reproduction, DON synthesis, and plant infection. Moreover, the Sgh1 is mainly localized to the nucleus. RNA-seq analysis has shownthat the expression of over 1100 genes and the splicing efficiency in over 300 introns were affected in the Δ*sgh1* mutant. Although the RS domain and all three of the RRM domains are important for the Sgh1 functions, only the RS domain is responsible for its nuclear localization. Finally, we verified that the Sgh1 interacts with the unique SR-specific kinase Srk1 in *F. graminearum* by the yeast-two hybrid (Y2H) and bimolecular fluorescence complementation (BiFC) assays. Taken together, our results have revealed that the Sgh1 regulates the fungal development, plant infection, and the pre-mRNA processing, and the RS domain regulates the function of the Sgh1 by modulating its nucleocytoplasmic shuttling.

## 1. Introduction

Fusarium head blight (FHB), or wheat scab, which is a destructive disease of wheat and barley worldwide, is caused by a homothallic filamentous ascomycete fungus called*Fusarium graminearum* [1]. In addition to wheat and barley, this pathogen also infects maize and causes Gibberella stalk rot and ear rot [2]. FHB not only causes severe yield losses, but it also produces multiple mycotoxins, including deoxynivalenol (DON) and zearalenone (ZEA) in the infected kernels [3,4]. The trichothecene mycotoxin DON was identified as the first virulent factor of the FHB fungus that has an inhibitory effect onthe protein synthesis in eukaryotic cells [5]. In spring, ascospores arising from sexual reproduction serve as the main source of primary infection [6,7]. The plant infection is initiated when the ascospores are dispersed and deposited onto the flowering wheat heads. The ascospores germinate and penetrate the wheat epidermal cells directly, or with specialized infection structures known as infection cushions [8]. After the initial colonization, the pathogen can spread from the infection site to neighboring florets and can cause severe symptoms under favorable environmental conditions [9].

In eukaryotic organisms, pre-mRNA splicing is an essential step of gene expression that is carried out by the spliceosome, which is a large dynamic RNA–protein complex comprising five SnRNPs (U1, U2, U4/U6, and U5) [10]. The spliceosome can recognize splicing signals and can catalyze the intron excision and exon ligation in order to produce mature mRNA [11]. This process is mainly regulated by various splicing factors, including heterogeneous nuclear ribonucleoproteins (hnRNPs) and serine/arginine-rich (SR) proteins [12]. The hnRNPs usually inhibit the splicing process by binding to the splicing silencer sequences and blocking the interaction between the pre-mRNA and the spliceosome [13]. Conversely, the SR proteins frequently promote RNA splicing as splicing activators by binding the splicing enhancer sequences in order to recruit the spliceosome to the nearby splice site of the pre-mRNA or antagonizing the effects of the hnRNPs [14,15]. The SR proteins play important roles in both constitutive and alternative splicing through multiple modes [16]. In addition, the SR proteins are also involved in mRNA export, RNA decay, and protein translation [17,18].

The typical SR proteins consist of a variable-length arginine and serine-rich (RS) domain at the C-terminus and at least one RNA recognition motif (RRM) at the N-terminus [19]. In general, the RS domains mediate diverse protein–protein and protein–RNA interactions, and RRM domains recognize the specific pre-mRNA sequence elements [16]. In addition, the SR protein-specific kinases (SRPKs) can phosphorylate the RS domain of the shuttling SR proteins, which is important for their subcellular localization and functions [17,20]. A total of 12 SR proteins have been identified in humans, which are named SRSF1–SRSF12 [15]. Metazoans and plants have a large number of SR proteins, whereas fungi generally contain 1–3 SR or SR-like proteins [21]. Only two SR proteins (Srp1 and Srp2) have been identified and characterized in fission yeast [22]. However, no typical SR protein, apart from three SR-like proteins (Npl3, Hrb1, and Gbp2), have been identified in *S. cerevisiae* [12]. 

Recently, two SR proteins (FgSrp1 and FgSrp2) were functionally characterized in *F. graminearum*. They play important roles in the vegetative growth, conidiation, DON biosynthesis, and plant infection [23,24]. In this study, we have identified an SR-like protein known as FGRAMPH1_01T26155, named Sgh1 (for ortholog of SR-like proteins Gbp2 and Hrb1). Although Gbp2/Hrb1 orthologs are conserved in filamentous ascomycetes, their functions in plant-pathogenic fungi are still unclear. In this study, we have not only determined the critical functions of Sgh1 in fungal development, pathogenicity, and pre-mRNA processing, but have also revealed the important functions of its RS and RRM domains.

## 2. Materials and Methods

### 2.1. Bioinformatics Analyses

The protein sequences of budding yeast paralogous Hrb1 and Gbp2 were downloaded from the Saccharomyces GenomeDatabase (SGD, http://www.yeastgenome.org, accessed on 18 May 2022). BLASP searches were performed in the database of *F. graminearum* at Ensembl Fungi (https://fungi.ensembl.org/Fusarium_graminearum/Info/Index, accessed on 18 May 2022) by using the Hrb1 and Gbp2 sequences, respectively. The FGRAMPH1_01T26155 (Sgh1) of *F. graminearum* was identified as the only ortholog of Hrb1/Gbp2 with an E-value lower than 1E-5. The best hits were then confirmed by reverse BLASTP search using the *F. graminearum* Sgh1 as the query. The Sgh1 orthologs from other representative fungi were obtained from NCBI using the BLASTP algorithm. A multiple alignment of the representative Sgh1 orthologs was generated using the program ClustalX V2.1 and shaded with the BoxShade V3.21. Identical and similar amino acid residues were shaded in black and gray, respectively. The conserved domains of Sgh1 were predicted using the Pfam database (http://pfam.xfam.org/, accessed on 18 May 2022). The putative nuclear localization signal (NLS) were predicted by the NLStradamus online software (http://www.moseslab.csb.utoronto.ca/NLStradamus/, accessed on 18 May 2022).

### 2.2. Fungal Strains and Culture Conditions

The wild-type strain PH-1, and all transformants used in this study, are listed in Table 1. All strains were routinely grown on complete medium (CM) agar at 25 °C. To determine the vegetative growth rate on CM solid medium, the diameters of colonies formed on 90 mm petri plates were measured after incubation for 3 days. Conidiation was assayed with 5-day-old CMC cultures [25] and sexual reproduction was performed on carrot agar plates, as previously described [26]. Protoplast preparation and fungal transformation were performed as previously described [25,27]. TB3 medium (0.3% casamino acids, 0.3% yeast extract, 20% sucrose, and 1.5% agar) with an addition of 300 μg/mL hygromycin B (CalBiochem, La Jolla, CA, USA) or 400 μg/mL G418 (MP Biochemicals, La Jolla, CA, USA) was used for transformant selection [28]. For DNAand RNA isolation, mycelia were harvested from the liquid YEPD (1% yeast extract, 2% peptone, 2% glucose) cultures by filtration through sterile miracloth.

### 2.3. Generation of Δsgh1 Mutants

To generate the Δ*sgh1* mutants, the split-marker approach was performed as previously described [29]. The 1.0-kb upstream and 0.8-kb downstream flanking fragments were amplified with primer pairs SGH1-1F/2R and SGH1-3F/4R, respectively. Two hygromycin phosphotransferase (*hph*) fragments (H1 and H2) were amplified with primer pairs HYG-F/HY-R and YG-F/HYG-R, respectively. Subsequently, the upstream and downstream fragments of *SGH1* were fused to corresponding fragments H1 and H2 of the *hph* gene by overlapping PCR and were transformed into PH-1 as previously described [30,31]. Transformants were picked from TB3 selection plates containing 300 μg/mL hygromycin B and were screened by PCR analysis for the deletion of the*SGH1* gene. Finally, putative Δ*sgh1* mutants were confirmed by Southern blot analysis using the DIG High Prime DNA Labeling and Detection Starter Kit I (Roche Applied Science, Mannheim, Germany) following the manufacturer’s instruction manual. The fragment that was amplified with primers SGH1-1F and SGH1-2R was labeled with digoxin (DIG) as probe A (Figure S3A). All primers used for PCR are listed in Table S1.

### 2.4. Generation of the SGH1-, SGH1^Δ^^RS^-, SGH1^ΔRRM1^-, SGH1^ΔRRM2^-, and SGH1^ΔRRM3^-GFP

All GFP fusion constructs were generated using the ClonExpress^®^ II One Step Cloning Kit (Vazyme, Nanjing, China). To generate the*SGH1*-GFP construct, the *SGH1* gene, with its native promoter, was amplified with primers SGH1-GF and SGH1-GR (Table S1). Subsequently, the PCR product was then cloned into the *Kpn* I/*Hind* III double-digested pKNTG vector to obtain the *SGH1*-GFP construct. To generate the*SGH1*^ΔRS^-GFP construct, two fragments that were amplified with primer pair SGH1-GF/DRS1R and DRS2F/SGH1-GR were fused by overlapping PCR. The resulting PCR product was then cloned into the *Kpn* I/*Hind* III double-digested pKNTG vector to gain the*SGH1*^ΔRS^-GFP construct. The same approach was used to generate constructs *SGH1*^ΔRRM1^-GFP, *SGH1*^ΔRRM2^-GFP, and *SGH1*^ΔRRM3^-GFP. All the primers used are listed in Table S1. All resulting GFP fusion constructs were confirmed by sequencing analysis.

### 2.5. Plant Infection and DON Production Assays

For plant infection assays, conidia harvested from 5-day-old CMC cultures were resuspended to a final concentration of 2 × 10^5^ spores/mL in sterile water. The flowering wheat heads of cultivar Xiaoyan22 were drop-inoculated with 10 µL conidial suspensions at the fifth spikelet from the base of the spike, as previously described [30], or with 10 µL sterile water as a mock control. The wheat heads with typical scab symptoms were examined at 14 days post-inoculation (dpi) and disease indexes were calculated by counting the number of symptomatic spikelets per spike, as previously described [32]. One-way ANOVA analysis, followed by Duncan’s multiple range test (*p* = 0.05), was used to calculate the significant differences. To observe the infection cushion, infected lemmas sampled at 2 dpi were fixed and coated with gold–palladium before examination with a scanning electron microscope, as previously described [33]. For assaying the infectious growth, infected rachis tissues were sampled at 5 dpi and embedded in Spurr resin after fixation and dehydration, as previously described [34]. Thick sections (1 μm) were then prepared and stained with 0.5% (wt/vol) toluidine blue before observation with an Olympus BX-53 microscope. For DON production assays, the liquid trichothecene biosynthesis (LTB) medium was used to induce DON synthesis. After incubation at 25 °C for 7 days, the DON concentrations in culture filtrates were assayed with a competitive ELISA-based DON detection plate Kit (Beacon Analytical Systems, Saco, ME, USA) [35]. The DON production assays were repeated three times.

### 2.6. Yeast Two-Hybrid and Bimolecular Fluorescence Complementation (BiFC) Assays

The split-ubiquitin yeast two-hybrid system (DUALsystems Biotech, Zurich, Switzerland) was performed to detect the protein–protein interactions. The *SGH1* gene was amplified by primersSGH1-NU/F and SGH1-NU/R, with cDNA of PH-1 as a template. The resulting PCR product was then cloned into an*EcoR* I-digested pPR3-N vector by using the ClonExpress^®^ II One Step Cloning Kit (Vazyme, Nanjing, China) as the prey construct. The same approach was performed to clone the ORF of the*SRK1* gene intoan*Nco* I-digested pDHB1 vector as thebait construct. The resulting bait and prey constructs, which were verified by sequencing analysis, were co-transformed into yeast strain NMY51. The yeast transformants isolated from the SD-Trp-Leu selection medium were assayed for their viability on SD-Trp-Leu-His-Ade medium and galactosidase activities with filter lift assays, as previously described [35]. To exclude the autoactivity of the examined bait (*SRK1*) and prey (*SGH1*) combination, the prey plasmid (pDHB1-*SRK1*) was co-transformed with an empty pPR3-N vector and the bait plasmid (pPR3-N-*SGH1*) with an empty pDHB1 vector. The resulting yeast transformants (pDHB1-*SRK1* + pPR3-N and pDHB1 + pPR3-N-*SGH1*) were examined for their viability on SD-Trp-Leu-His-Ade medium and galactosidase activities with filter lift assays.

To further confirm the interaction between Sgh1 and Srk1, we performed the BiFC assays as previously described [27]. Constructs*SRK1*^ΔS^-NYFP and *SGH1*-CYFP were generated by cloning *SRK1*^ΔS^and *SGH1* into pHZ65 and pHZ68 vectors, respectively, as previously described [27]. The resulting fusion constructs were verified by sequencing analysis and then co-transformed into the protoplasts of PH-1. The transformants expressing both *SRK1*^ΔS^-NYFP and *SGH1*-CYFP were isolated from TB3 selection plates containing 300 μg/mL hygromycin B and 400 μg/mL G418, and then further confirmed by PCR analysis. YFP signals were examined using aZeiss LSM880 confocal microscope (Carl Zeiss, Jena, Germany). The transformants of PH-1 expressing *SRK1*-NYFP + CYFP or NYFP + *SGH1*-CYFP were used as negative controls. All the primers used are listed in Table S1.

### 2.7. RNA-Seq Analysis

Mycelia of the wild-type strain PH-1 and Δ*sgh1* mutant were collected from 12 h CM cultures. Total RNA samples were extracted with the Oligotex mRNA mini kit (Qiagen, Germany). Two biological replicates for each strain were prepared. Library construction and sequencing with an Illumina Hiseq-2500 sequencer with a 2 × 150 bp paired-end read mode were performed at Novogene Bioinformatics Institute (Beijing, China). The RNA-seq reads of PH-1 and the Δ*sgh1* mutant were mapped to the reference genome of PH-1 via Hisat2 [36]. The feature counts were used to calculate the number of reads aligned to each predicted transcript [37]. The differential expression genes (DEGs) between the PH-1 and Δ*sgh1* mutant were analyzed by edgeRun [38].The up- or down-regulation was defined as a fold change of >2 or <0.5, with significance determined at *p* < 0.05. Differential alternative events between PH-1 and the Δ*sgh1* mutant were detected as previously described [24].

The RNA-seq data of vegetative hyphae and perithecia of PH-1 were generated and deposited in the NCBI SRA database under accession numbers SRS1044675 and SRS1044677 [39]. The RNA-seq data of infected wheat heads were deposited under accession numbers SRR8568982–SRR8568984 and SRR8569386–SRR8569394 [33]. These published RNA-seq data were downloaded from the NCBI SRA database. The Trimmomatic was used to remove the low-quality reads of the RNA-seq data. The resulting high-quality reads were aligned to the PH-1 reference genome using Hisat2 with its two-step algorithm. The feature counts were used to calculate the count of reads aligned to each gene. The gene expression counts were normalized using the TPM (transcripts permillion) method and the *SGH1* gene expression levels in different samples were estimated by TPM counts.

### 2.8. CFW and DAPI Staining

To visualize the cell walls, septa, and nuclei clearly, conidia and hyphae were incubated with 10 μg/mL CFW and 20 μ g/mL 4′, 6-diamidino-2-phenylin-dole (DAPI) (Sigma) for 5–10min in the dark at 25 °C, as previously described [40,41]. Subsequently, the samples were observed under UV light using a Zeiss LSM880 confocal microscope (Carl Zeiss, Jena, Germany).

### 2.9. Quantification of Nuclear/Cytoplasmic Intensity Ratio of Fluorescence

To determine the nuclear/cytoplasmic fluorescence intensity ratio of Sgh1-GFP or Sgh1^∆RS^-GFP, the conidia were imaged by a Zeiss LSM 880 microscope (Carl Zeiss, Jena, Germany) with an objective Plan-Apochromat 63× (NA = 1.4) oil immersion and illumination of 488 nm. To analyze the images, the region of interest (ROI) was manually drawn in the nucleus of an individual cell by Fiji/ImageJ (National Institutes of Health). One reference region of identical size was drawn within the cytoplasm in the same cell. Mean fluorescence intensities were measured for these two regions of each cell using Fiji/ImageJ. The relative nuclear enrichment was calculated as the ratio between mean nuclear and cytoplasmic fluorescence intensities. More than 30 conidia were examined for each strain. Data of replicates were pooled before significance testing. One-way ANOVA, followed by Duncan’s multiple range test (*p* = 0.05), was used to test for significance.

## 3. Results

### 3.1. FGRAMPH1_01T26155 Encodes a Conserved SR-like Protein in Filamentous Ascomycetes

In *F. graminearum*, two SR proteins, FgSrp1 and FgSrp2, have been well characterized [23,24]. In this study, we identified an SR-like protein known as Sgh1 (FGRAMPH1_01T26155) in *F. graminearum*, which is orthologous to Hrb1 and Gbp2, two paralogs from *S. cerevisiae*. The *SGH1*gene encodes a 489-aa protein that shares only 24.5% and 25.2% identities in amino acid sequences with budding yeast Hrb1 and Gbp2, respectively. However, we also found that there is only one Hrb1/Gbp2-orthologous protein in other fungi, including *F. graminearum*, *Fusarium oxysporum*, *Magnaporthe oryzae*, *Aspergillus nidulans*, *Ustilago maydis,*and *Candida albicans*. The multiple sequence alignment analysis has shown that the Hrb1/Gbp2 orthologs are conserved in filamentous ascomycetes (Figure S1). Unlike the structure organization of yeast SR or SR-like proteins Srp1, Srp2, and Npl3, the Sgh1 has three RRM domains (RRM1: 105–176 aa, RRM2: 228–302 aa, and RRM3: 392–459 aa) at the C-terminal region and one RS domain (15–80 aa) at the N-terminal region (Figure S1, Figure S2A). One putative nuclear localization signal (NLS, 15–46 aa) was predicted in the RS domain (Figure S1) by the NLStradamus online software (http://www.moseslab.csb.utoronto.ca/NLStradamus/, accessed on 18 May 2022) [42]. Moreover, Sgh1 has nine putative serine phosphorylation sites within the RS domain and an RGG-box motif containing five RGG repeats between the RRM2 and RRM3 domains (Figure S1). According to our previous RNA-seq data [33,39], the *SGH1* is expressed in mature conidia and 12 h germlings, and during sexual development and infectious process, but the expression level was higher in the early stage of sexual reproduction (Figure S2B).

### 3.2. The Δsgh1 Mutant Is Defective in Vegetative Growth, Conidiogenesis, and Sexual Reproduction

**Figure 1:**
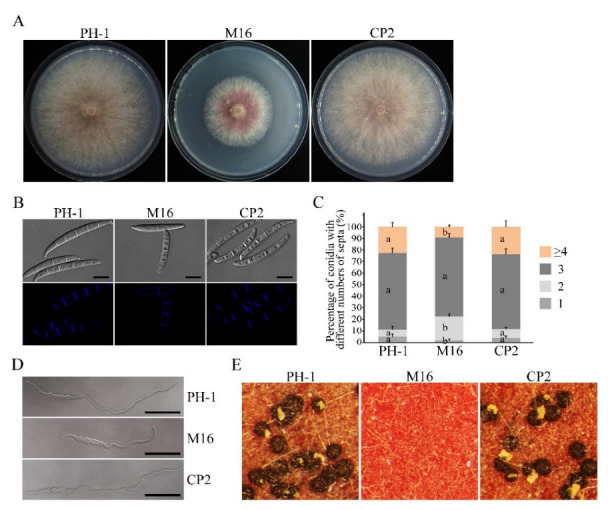
Defects of the Δ*sgh1* mutant in growth, conidiogenesis, conidial germination, and sexual reproduction. (**A**).The wild type (PH-1), Δ*sgh1* mutant (M16), and complemented transformant Δ*sgh1*/*SGH1*-GFP (CP2) were cultured on CM plates for three days. (**B**). Conidia of the same set of strains were examined by differential interference contrast (DIC) microscopy. Bar = 10 μm. (**C**). Percentage of conidia with different numbers of septa in PH-1, M16, and CP2 (more than 300 conidia were examined for each strain). The error bars represent the standard deviations. The different letters indicate statistically significant differences by Duncan’s multiple range test (*p* = 0.05). (**D**). The germlings of the same set of strains were examined for defects in germination and germ tube growth by DIC microscopy after incubation in YEPD for 6 h. Bar = 50 μm. (**E**). Perithecium formation on carrot agar cultures of the labeled strains was examined at 8 dpf.

In order to investigate the function of the*SGH1* gene in *F. graminearum*, we generated the Δ*sgh1* deletion mutants with the split-marker approach, as previously described [29]. Three hygromycin-resistant transformants (Table 1), which were verified by PCR analysis, were further confirmed by Southern blot analysis (Figure S3). When hybridized with the probe A, which was amplified with primers SGH1-1F and SGH1-2R, 1.3 kb and 2.5 kb *Xho* I bands were detected in the wild-type PH-1 and Δ*sgh1*mutants, respectively (Figure S3), indicating that correct gene replacement events occurred in the mutants Δ*sgh1*-6, 14, and 16. These three Δ*sgh1*mutants showed the same phenotypes in theircolony morphology, vegetative growth, conidiation, and sexual reproduction (Figure S4); however, only the Δ*sgh1*-16 mutant was selected for further characterization. Careful examinations have revealed that the Δ*sgh1* mutant was reduced by approximately 40% in growth rate on the CM plates when compared to the wild-type PH-1 (Figure 1A and Table 2). In addition, the Δ*sgh1* mutant was also reduced by 72% in conidiation (Table 2) and it wasdefective in conidial morphology (Figure 1B). The conidia of the Δ*sgh1* mutant had fewer septa (Figure 1B,C) and produced shorter germlings than those of PH-1 at 6 h post-incubation (hpi) (Figure 1D). For complementation assays, the *SGH1*-GFP fusion construct with its native promoter region was generated and transformed into the Δ*sgh1* mutant. In the resulting Δ*sgh1*/*SGH1*-GFP transformant CP2 (Table 1), the defects of the Δ*sgh1* mutant in vegetative growth and conidiogenesis were restored (Figure 1 and Table 2). These results have revealed that the *SGH1* is important for vegetative growth and conidiogenesis.

**Table 1:** The wild-type and mutant strains of *F. graminearum* used in this study.

Strain	Brief Description	Reference
PH-1	Wild-type strain	[43]
M6, M14, M16	*SGH1* deletion mutant of PH-1	This study
CP2	Transformant of M16 expressing *SGH1*-GFP construct	This study
DRS2	Transformant of M16 expressing *SGH1*^ΔRS^-GFP construct	This study
DRRM1-1	Transformant of M16 expressing *SGH1*^ΔRRM1^-GFP construct	This study
DRRM2-2	Transformant of M16 expressing *SGH1*^ΔRRM2^-GFP construct	This study
RRRM3-2	Transformant of M16 expressing *SGH1*^ΔRRM3^-GFP construct	This study
BFSS-5	Transformant of PH-1 expressing *SRK1*^ΔS^-YFPN and *SGH1*-YFPC constructs	This study
DSSG-3	Transformant of Δ*srk1* mutant expressing *SGH1*-GFP construct	This study

Ascospores had been identified as the primary inoculum for epidemics of wheat scab disease. Thus, we investigated the sexual reproduction of theΔ*sgh1* mutant on carrot agar medium. The Δ*sgh1* mutant failed to produce any proto-perithecia or perithecia on mating plates at eight days post-fertilization (dpf), while the PH-1 formed numerous black, mature perithecia under the same conditions (Figure 1E and Table 2). In the complemented transformant Δ*sgh1*/*SGH1*-GFP, the defect in sexual reproduction was also restored (Figure 1E and Table 2). These data indicate that *SGH1* is indispensable for the initial phase of sexual reproduction.

**Table 2:** Defects of the Δ*sgh1* mutant in growth, conidiation, and DON production.

Strain	Growth Rate	Conidiation	Perithecia Formation	DON (μg/g) ^d^
	(mm/d) ^a^	(10^5^ Spores/mL) ^b^	(Perithecia/cm^2^) ^c^	
PH-1	7.5 ± 0.1 ^a^	24.41 ± 2.79 ^a^	665.7 ± 88.8 ^a^	1436.36 ± 19.53 ^a^
M16	4.5 ± 0.1 ^b^	6.81 ± 1.53 ^b^	0 ^b^	585.91 ± 55.50 ^b^
CP2	7.5 ± 0.3 ^a^	25.87 ± 2.6 ^a^	676.0 ± 83.7 ^a^	1497.24 ± 23.76 ^a^
DRS2	7.4 ± 0.1 ^a^	21.86 ± 1.29 ^a^	708.8 ± 123.4 ^a^	1381.97 ± 284.16 ^ac^
DRRM1-1	7.3 ± 0.2 ^a^	22.41 ± 0.65 ^a^	713.4 ± 93.1 ^a^	1191.93 ± 209.51 ^c^
DRRM2-2	7.4 ± 0.2 ^a^	20.93 ± 0.22 ^a^	670.3 ± 80.8 ^a^	911.96 ± 251.08 ^c^
DRRM3-2	5.6 ± 0.1 ^c^	11.16 ± 0.61 ^c^	340.2 ± 85.9 ^c^	637.99 ± 38.06 ^b^

The means ± SE were calculated from the results of three independent experiments.Different letters were used to mark the statistically significant differences based on one-way ANOVA analysis, followed by Duncan’s multiple range test (*p* = 0.05). ^a^ The average growth rate was measured as the daily expansion of the colony radius after incubation on CM for three days. ^b^ The conidiation in 5-day-old CMC liquid cultures. ^c^ The 7-dpf selfing cultures of the indicated strains were counted to calculate the number of perithecia per cm^2^. ^d^ The DON production in the LTB cultures.

### 3.3. SGH1 Plays a Critical Role in Plant Infection

**Figure 2:**
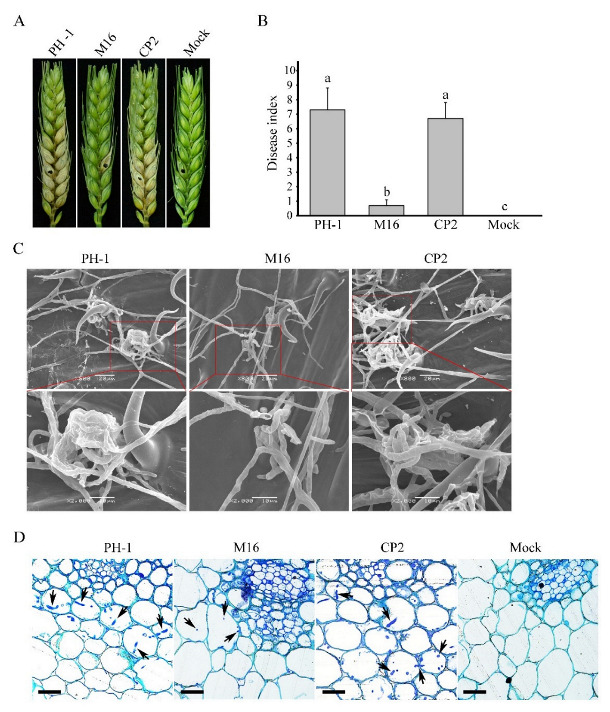
Assays for the function of Sgh1 in pathogenesis. (**A**). Wheat heads inoculated with the wild-type strain (PH-1), the Δ*sgh1* mutant (M16), and the complemented transformant Δ*sgh1*/*SGH1*-GFP (CP2) were examined for head blight symptoms at 14 dpi. Sterile distilled water was mock-inoculated as a negative control. The black dots mark the inoculated spikelets. (**B**). Mean and standard deviation (SD) of the disease index of the same set of strainswere estimated from three independent experiments, with at least 10 infected wheat heads in each experiment. The different letters indicate significant differences based on ANOVA analysis, followed by Duncan’s multiple range test (*p* = 0.05). (**C**). Infection cushions formed by the indicated strains on wheat lemma at 2 dpi were examined by SEM under ×800 and ×2000 magnification. The representative micrographs show the defect in infection cushion formation in the Δ*sgh1* mutant. Bar = 20 μm. (**D**). Thick sections of the rachis tissues right below the inoculated spikelets were examined at 5 dpi in the same set of strains. The invasive hyphae were marked with arrows. Bar = 100 μm.

We have also assayed the defects of the Δ*sgh1* mutant in plant infection. The conidia suspensions of the PH-1 and the Δ*sgh1* mutant were drop-inoculated in the spikelets of the flowering wheat heads. At 14 dpi, the wild-type strain PH-1 caused typical scab symptoms in the inoculated and nearby spikelets. In contrast, the Δ*sgh1* mutant only caused symptoms that were limited to the inoculated kernels, but they never spread to the neighboring spikelets (Figure 2A). In the mock control, the wheat head did not show any scab symptoms. We carefully measured the disease index by counting the diseased spikelets per wheat head. The average disease index of the Δ*sgh1* mutant, the PH-1, and the mock was 0.7, 7.3, and 0, respectively (Figure 2B). Since DON is an important virulence factor in *F. graminearum* [5], we also investigated the effect of *SGH1* deletion on the DON production. In LTB cultures, the concentration of DON that was produced by the PH-1 and the Δ*sgh1* mutant were 1436.36 μg/g and 585.91 μg/g, respectively (Table 2), indicating that the Δ*sgh1* mutant was reduced by approximately 60% in DON biosynthesis. In the complemented transformant Δ*sgh1*/*SGH1*-GFP CP2, the defects in the pathogenicity and the DON production were restored (Figure 2A,B and Table 2). These results indicate that the *SGH1*gene plays an important role in the pathogenicity and DON production in *F. graminearum*.

In order to further characterize the defects of the Δ*sgh1* mutant in plant infection, we examined the formation of the infection cushion in the infected wheat heads by scanning electron microscopy (SEM). The wild-type PH-1 formed infection cushions on wheat lemma at 2 dpi, which facilitate its penetration into the plant tissue, while the Δ*sgh1* mutant failed to produce typical infection cushions (Figure 2C). The infection cushions of the Δ*sgh1* were less complex, indicating that the *Sgh1* plays an important role in the infection cushion formation. When they were examined for the infectious growth in the rachis, which is essential for the pathogen spreading in wheat heads, abundant invasive hyphae were observed in the samples that were inoculated with PH-1 at 5 dpi (Figure 2D). However, under the same conditions, the invasive hyphae were rarely observed in the rachis tissues below the infected spikeletsthat were inoculated with the Δ*sgh1* mutant (Figure 2D). By contrast, the mock-inoculated florets did not exhibit any invasive hyphae in rachis tissue. Moreover, the defects of the Δ*sgh1* mutant in the infection cushion formation and the invasive growth were suppressed in the complemented transformant Δ*sgh1*/*SGH1*-GFP (Figure 2C,D). Therefore, the defects of theΔ*sgh1* mutant in the infection cushion formation and the invasive growth may contribute to its reduced pathogenicity.

### 3.4. The Δsgh1 Deletion Mutant Showed Increased Sensitivity to Osmotic and Cell Wall Stresses

**Figure 3:**
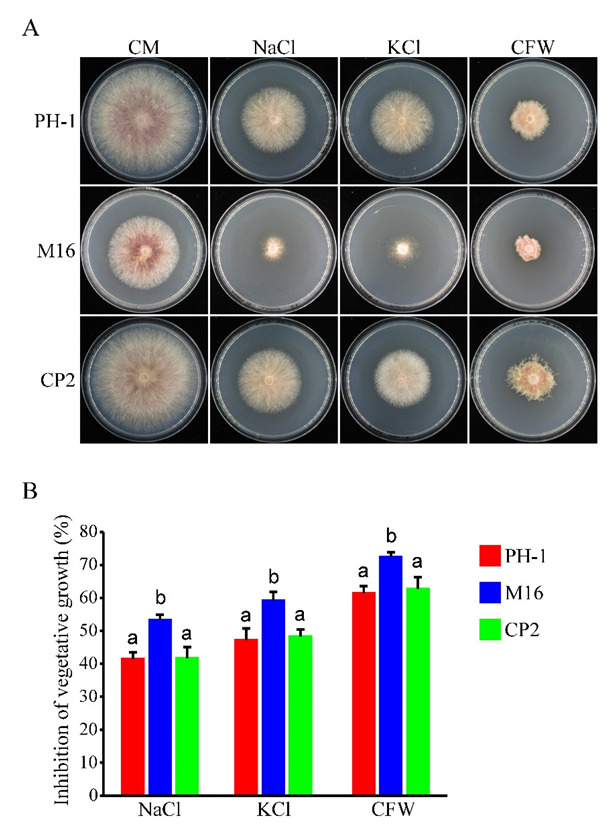
The Δ*sgh1* mutant exhibited sensitivity to osmotic and cell wall stresses. (**A**). The wild type (PH-1), Δ*sgh1* mutant (M16), and complemented transformant Δ*sgh1*/*SGH1*-GFP (CP2) were cultured on CM plates with or without 1 M NaCl, 1 M KCl, and 200 μg/mL CFW for 3 days. (**B**). The mean and standard deviation of mycelial growth inhibition of each strain under each treatment were estimated with data from three biological replicates. The different letters indicate significant differences.

In order to determine whether the Δ*sgh1* mutant has defects in stress responses, we assayed its growth rate on CM plates that were supplemented with 1 M NaCl, 1 M KCl, and 200 μg/mL Calcofluor White (CFW). In the presence of NaCl, KCl, or CFW, the growth inhibition rates of theΔ*sgh1* mutant were significantly higher than those of the PH-1 (Figure 3), indicatingthat the Δ*sgh1* mutant was sensitive to osmotic and cell wall stresses. Moreover, the defects of the Δ*sgh1* mutant in sensitivity to osmotic and cell wall stresses were also rescued in the complemented transformant Δ*sgh1*/*SGH1*-GFP (Figure 3). These results indicate that the Sgh1 is involved in the responses against osmotic and cell wall stresses.

### 3.5. Subcellular Localization of Sgh1-GFP Fusion Protein

**Figure 4:**
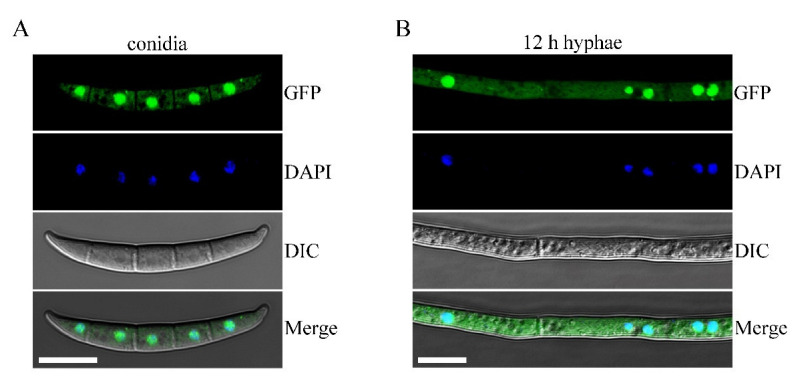
Subcellular localization of Sgh1-GFP fusion protein. (**A**). Fresh conidia harvested from thecomplemented transformant Δ*sgh1*/*SGH1*-GFP (CP2) were stained with 4,6-diamidino-2-phenylindole (DAPI) and examined witha Zeiss LSM880 confocal microscope. GFP signals were present in both nuclei and cytoplasm. Bar = 10 μm. (**B**). The 12 h hyphae of CP2 transformant were observed by DIC and epifluorescence microscopy. Bar = 10 μm.

In a previous study, the transformant Δ*sgh1*/*SGH1*-GFP displayed the same growth rate, conidiation, sexual reproduction, pathogenicity, and stress responses as the wild-type PH-1 (Figure 1, Figure 2 and Figure 3 and Table 2), indicating that the *SGH1*-GFP fusion construct completely restored the defects of the Δ*sgh1* mutant. Since the *SGH1*-GFP fusion construct is functional, we examined the subcellular localization of Sgh1-GFP. Under epifluorescence microscopy, the Sgh1-GFP signals were present in both the cytoplasm and the nuclei in fresh conidia and 12 h hyphae, which was confirmed by staining with 4, 6-diamidino-2-phenylindole (DAPI) (Figure 4). However, the nuclei had stronger GFP signals than the cytoplasm (Figure 4), indicating that the majority of Sgh1-GFP fusion proteins is localized to the nucleus.

### 3.6. Sgh1 Regulates RNA Splicing and Gene Transcription

**Figure 5:**
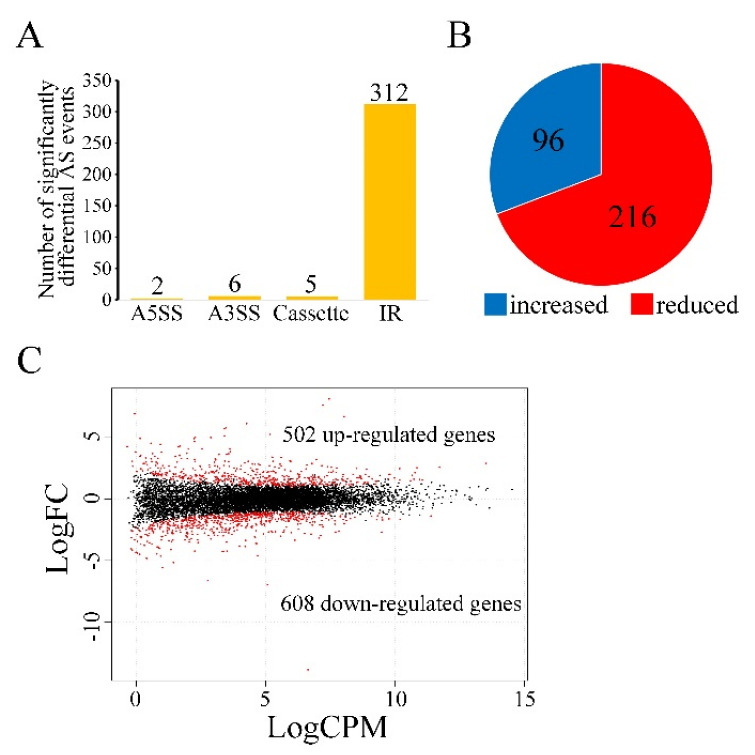
Differential alternative splicing (AS) and transcription in the Δ*sgh1* mutant. (**A**). The number of significantly differential AS events in the Δ*sgh1*mutant relative to the wild−type PH−1. Types of AS events include A5SS (Alternative 5′ splice site), A3SS (Alternative 3′ splice site), Cassette (cassette exon), and IR (intron retention). (**B**). Percentage of IR events with increased or reduced splicing efficiency in the Δ*sgh1* mutant. (**C**). MAplot showing the log2-fold change (logFC) of individual genes plotted with the average expression strength (logCPM) in the Δ*sgh1*mutant compared to the wild type. The numbers of up-regulated and down-regulated genes were calculated with data from two biological replicates.

In order to determine the functions of Sgh1 in RNA splicing and gene transcriptional regulation in *F. graminearum*, we performed an RNA-seq analysis with RNA samples that were isolated from the vegetative hyphae of the PH-1 and the Δ*sgh1* mutant from 12 hpi CM cultures. In comparison to the PH-1, 325 significantly differential alternative splicing (AS) events were detected in the Δ*sgh1* mutant (Figure 5A, Dataset S1), indicating that the Sgh1 regulates the RNA splicing of a subset of genes. Among them, intron retention (IR) made up the vast majority of AS events that were detected (Figure 5A), accounting for approximately 96% of the total AS events (Dataset S1). Further analysis has revealed that 96 and 216 of IR events with increased and reduced RNA splicing efficiency were detected, respectively (Figure 5B, Dataset S1), indicating that Sgh1 plays both positive and negative roles in RNA splicing. When compared with the PH-1, 1110 differentially expressed genes (502 up-regulated and 608 down-regulated) were detected in the Δ*sgh1* mutant (Figure 5C, Dataset S2), accounting for 10.4% of the total expressed genes. A number of genes that are required for vegetative growth, sexual reproduction, and pathogenicity were significantly down-regulated in the Δ*sgh1* mutant, including two protein kinases genes,*FGK3* (FGRRES_07329) and *FgYAK1* (FGRRES_05418) [26], five transcriptional factors, including *FgMCM1* (FGRRES_08696), FGRRES_10470, FGRRES_07133, FGRRES_08572, and FGRRES_10716 [44,45], and the *ACL2* gene encoding an ATP citrate lyase [46]. These results indicate that the Sgh1 is important for the regulation of RNA splicing and gene transcription.

### 3.7. The RS Domain Is Important for Both Functions and Nuclear Localization of Sgh1

**Figure 6:**
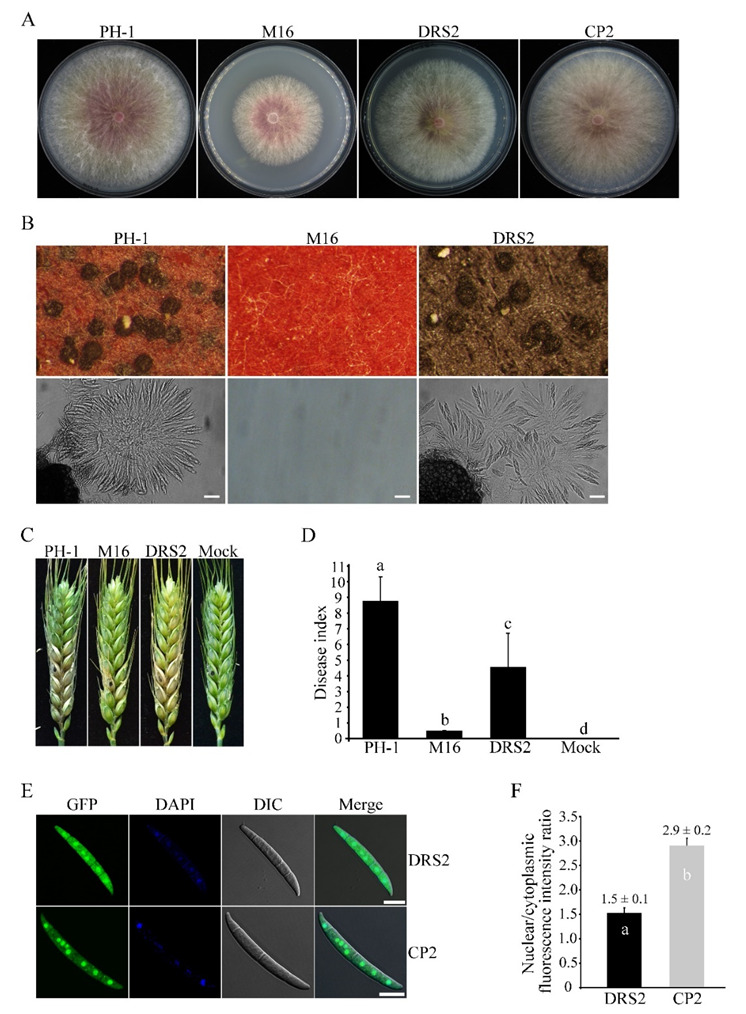
Functional characterization of the RS region in Sgh1 protein. (**A**). Three-day old CM cultures of the wild-type PH-1, Δ*sgh1* mutant (M16), Δ*sgh1*/*SGH1*^ΔRS^-GFP transformant (DRS2), and complemented transformant Δ*sgh1*/*SGH1*-GFP (CP2). (**B**). Sexual reproduction assays with PH-1, M16, and DRS2 at 8 dpf. Bar = 50 μm. (**C**). Flowering wheat heads inoculated with the indicated strains were examined for head blight symptoms at 14 dpi. Sterile distilled water was mock-inoculated as a negative control. The black dots mark the inoculated spikelets. (**D**). The bar chart shows the disease indexes of the indicated strains at 14 dpi. Each data point represents the mean from three independent experiments with at least 10 infected wheat heads in each experiment. The error bars indicate standard deviations. The different letters indicate significant differences. The significant differences were analyzed by Duncan’s multiple range test (*p* = 0.05). (**E**). Fresh conidia harvested from transformants Δ*sgh1*/*SGH1*-GFP (CP2) and Δ*sgh1*/*SGH1*^ΔRS^-GFP (DRS2) were stained with DAPI and examined witha Zeiss LSM880 confocal microscope. Bar = 10 μm. (**F**). The bar graph shows the nuclear/cytoplasmic intensity ratios of Sgh1^ΔRS^-GFP and Sgh1-GFP in conidia, respectively (more than 30 conidia were examined). One-way ANOVA, followed by Duncan’s multiple range test (*p* = 0.05), was used to test for significance. The different letters indicate significant differences.

In typical SR proteins, the RS domain plays an important role in subcellular distribution or protein function [47]. In order to determine the RS domain function in Sgh1, we generated the *SGH1*^ΔRS^-GFP construct, in which the RS domain was deleted, and transformed it into theΔ*sgh1* mutant. The resulting transformant Δ*sgh1*/*SGH1*^ΔRS^-GFP (Table 1) showed a slightly reduced growth rate on the CM plate and no detectable defect in sexual development (Figure 6A,B and Table 2). However, the transformant Δ*sgh1*/*SGH1*^ΔRS^-GFP was significantly reduced in pathogenicity in comparison to the PH-1, but its disease index was higher than that of the original Δ*sgh1* mutant (Figure 6C,D and Table 2). By contrast, the disease index of the mock control was 0. When they were examined by fluorescent microscopy, the Sgh1^ΔRS^-GFP and the Sgh1-GFP were localized in both the nucleus and the cytoplasm of fresh conidia. However, more Sgh1^ΔRS^-GFP signals were detected in the cytoplasm than Sgh1-GFP signals (Figure 6E). Further analysis showed that the nuclear versus cytoplasmic (N/C) intensity ratios of the Sgh1-GFP and the Sgh1^ΔRS^-GFP were 2.9 and 1.5, respectively (Figure 6F), indicating that the deletion of the RS domain impairs the nuclear localization of Sgh1. These results indicate that the RS domain is important for both the functions and the nuclear localization of Sgh1.

### 3.8. Functional Characterization of the Three RRM Domains of Sgh1

**Figure 7:**
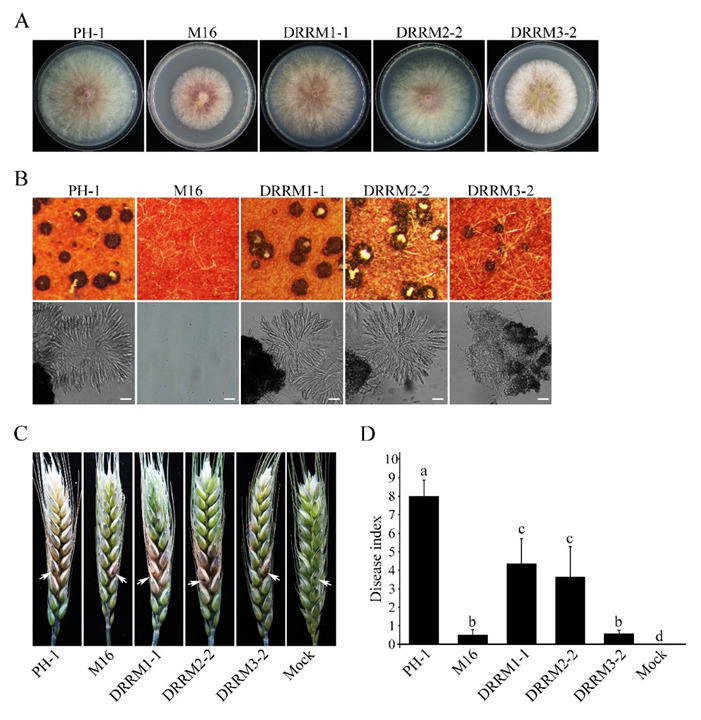
Functional characterization of the three RNA recognition motifs in Sgh1. (**A**). Three-day old CM cultures of the wild-type PH-1, Δ*sgh1* mutant (M16), and transformants Δ*sgh1*/*SGH1*^ΔRRM1^-GFP (DRRM1-1), Δ*sgh1*/*SGH1*^ΔRRM2^-GFP (DRRM2-2), and Δ*sgh1*/*SGH1*^ΔRRM3^-GFP (DRRM3-2). (**B**). Mating cultures of the same set of strains were examined for perithecia with ascospore cirrhi (upper panels) and asci from crushed perithecia (lower panels) at 8 dpf. Bar = 50 μm. (**C**). Flowering wheat heads inoculated with the indicated strains were examined for head blight symptoms at 14 dpi. Sterile distilled water was mock-inoculated as a negative control. Arrows mark the inoculated spikelets. (**D**). The bar chart shows the disease indexes of the indicated strains at 14 dpi. Each data point represents the mean from three independent experiments. The error bars indicate standard deviations. The different letters indicate significant differences by Duncan’s multiple range test (*p* = 0.05).

Besides the RS domain, the Sgh1 has three C-terminal RRM domains that are conserved among its orthologs in filamentous ascomycetes. We generated mutant alleles of *SGH1*, in which the RRM1, RRM2, or RRM3 domains were deleted, and transformed them into the Δ*sgh1* mutant, respectively. The transformants of the Δ*sgh1* expressing the *SGH1*^ΔRRM1^- and *SGH1*^ΔRRM2^-GFP alleles (Table 1) were slightly reduced in vegetative growth (Figure 7A and Table 2) but had no detectable defects in sexual reproduction (Figure 7B and Table 2). In addition, the Δ*sgh1*/*SGH1*^ΔRRM1^-GFP and Δ*sgh1*/*SGH1*^ΔRRM2^-GFP transformants showed significantly reduced pathogenicity on the wheat heads and the DON production in the LTB culture (Figure 7C,D and Table 2). However, the transformant Δ*sgh1*/*SGH1*^ΔRRM3^-GFP (Table 1) showed more severe defects in the vegetative growth, pathogenicity, and DON production than the transformants Δ*sgh1*/*SGH1*^ΔRRM1^-GFP and Δ*sgh1*/*SGH1*^ΔRRM2^-GFP(Figure 7A,C,D and Table 2). Moreover, the transformant Δ*sgh1*/*SGH1*^ΔRRM3^-GFP produced fewer perithecia that appeared smaller in size and failed to produce ascus or ascospore (Figure 7B and Table 2). These results indicate that the RRM3 domain is more important for the full function of Sgh1 than the RRM1 and RRM2 domains.

We also determined the roles of these three RRM domains that are described above in the subcellular localization of Sgh1. In the transformants of Δ*sgh1*/*SGH1*^ΔRRM1^-GFP, Δ*sgh1*/*SGH1*^ΔRRM2^-GFP, and Δ*sgh1*/*SGH1*^ΔRRM3^-GFP, the GFP signals were mainly localized in the nucleus, similar to the localization pattern of the wild-type Sgh1-GFP in the complemented transformant Δ*sgh1*/*SGH1*-GFP (Figure S5). Therefore, the deletion of individual RRM domains does not affect the subcellular localization of the Sgh1-GFP.

### 3.9. Sgh1 Physically Interacts with the SR Protein-Specific Kinase Srk1

**Figure 8:**
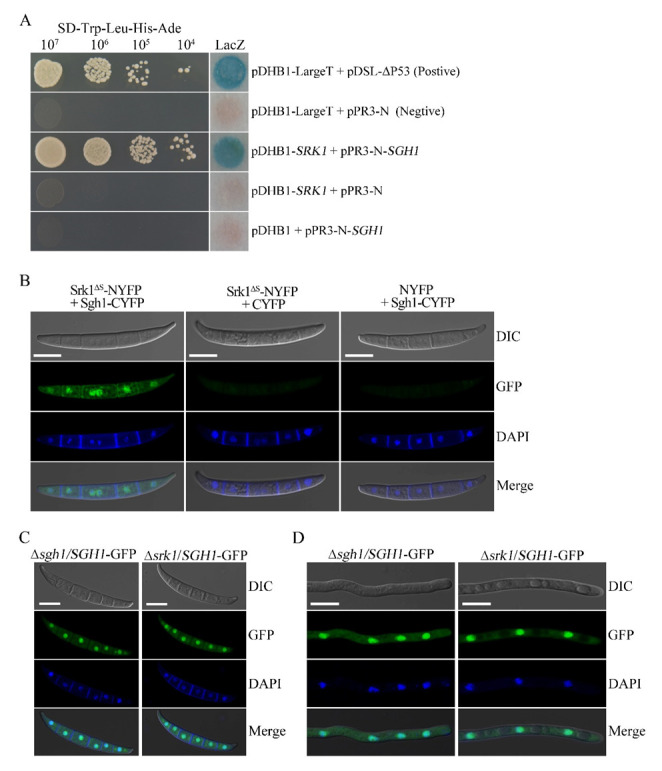
The Srk1 interacts with Sgh1 but does not affect its subcellular localization. (**A**). Yeast transformants containing the Srk1 bait and Sgh1 prey constructs grew on SD-Trp-Leu-His-Ade plates and displayed beta-galactosidase (LacZ) activities. Positive control, pDHB1-LargeT + pDSL-∆P53; Negative control, pDHB1-LargeT+pPR3-N. (**B**). Conidia of the transformants expressing the *SRK1*^ΔS^-NYFP and *SGH1*-CYFP fusion constructs were examined by epifluorescence microscopy. Transformants of PH-1 expressing *SRK1*-NYFP + CYFP or NYFP + *SGH1*-CYFP were used as the negative controls. No YFP signal was observed in these negative controls. Bar = 10 μm. (**C**). Fresh conidia of Δ*sgh1*/*SGH1*-GFP and Δ*srk1*/*SGH1*-GFP transformants were examined witha Zeiss LSM880 confocal microscope. Bar = 10 μm. (**D**). The 12 h germlings of Δ*sgh1*/*SGH1*-GFP and Δ*srk1*/*SGH1*-GFP transformants were examined witha Zeiss LSM880 confocal microscope. Bar = 10 μm.

In *F. graminearum*, the Srk1 is the unique SR protein-specific kinase that is orthologous to the budding yeast Sky1 and human SRPK1 [27]. In order to test whether the Sgh1 interacts with the Srk1, we performed the split-ubiquitin-based yeast two-hybrid assays. The *SGH1* prey and the *SRK1* bait constructs were co-transformed into the NMY51 yeast strain. In order to exclude the autoactivity, pDHB1-*SRK1* + pPR3-N and pDHB1 + pPR3-N-*SGH1* were co-transformed into the NMY51 yeast strain, respectively. The resulting yeast transformants expressing both the *SRK1* bait and the*SGH1* prey constructs can grow on an SD-Trp-Leu-His-Ade plate and display β-galactosidase (LacZ) activities in the colony lift filter assays (Figure 8A). We have also shown that the *SGH1* prey and the *SRK1* bait constructs had no autoactivity (Figure 8A). Therefore, the Sgh1 interacted with the Srk1 kinase in *F. graminearum*. In order to further verify the interaction between the Sgh1 and the Srk1, we employed the bimolecular fluorescence complementation (BiFC) assays. The *SRK1*^ΔS^-YFPN construct, in which the spacer domain was deleted, was generated in our previous study [27] and was co-transformed with *SGH1*-YFPC into the PH-1 strain. In the resulting transformant BFSS-5 (Table 1), YFP signals were observed in the nucleus (Figure 8B). These results indicate that the Sgh1 physically interacts with theSrk1 kinase in *F. graminearum*.

### 3.10. Deletion of SRK1 Kinase Does Not Affect the Subcellular Localization of Sgh1-GFP

In budding yeast and humans, the SR protein kinases regulate the nuclear localization of shuttling SR proteins by phosphorylation [48]. The Srk1 is the unique SR protein-specific kinase in *F. graminearum* [27]. In order to determine whether the nuclear localization of Sgh1 is dependent on Srk1 in *F. graminearum*, we transformed the*SGH1*-GFP construct into the Δ*srk1* mutant. When they were examined by epifluorescence microscopy, the Sgh1-GFP signals were mainly observed in the nucleus in the conidia or the 12 h hyphae of both the Δ*srk1*/*SGH1*-GFP and Δ*sgh1*/*SGH1*-GFP transformants, and no obvious difference was detected (Figure 8C,D). These results indicate that the Srk1 is dispensable for the nuclear localization of the Sgh1 in *F. graminearum*.

## 4. Discussion

In our previous study, two SR proteins, FgSrp1 and FgSpr2, were functionally characterized in *F. graminearum* [24]. In this study, we identified an SR-like protein, which is orthologous to budding yeast paralogous Gbp2 and Hrb1. Interestingly, only one Gbp2/Hrb1 ortholog is present in *F. graminearum*. The typical SR proteins have one or two RRM domains and one C-terminal RS domain, whereas the Gbp2/Hrb1 orthologs have an N-terminal RS domain followed by three RRM domains. To our knowledge, this is the first report of the functional characterization of Gbp2/Hrb1 ortholog in plant-pathogenic fungi.

In *F. graminearum*, the Δ*sgh1*deletion mutant was reduced by 40% in vegetative growth. Although Hrb1 orthologs are well conserved in filamentous ascomycetes, only the SNXA (ortholog of budding yeast Gbp2/Hrb1) had been characterized in *A. nidulans*. The *snxA* deletion mutant showed a growth defect and a cold-sensitive phenotype [49]. Therefore, the Gbp2/Hrb1 orthologs in filamentous ascomycetes may have a conserved function in vegetative growth. The sexual development of the Δ*sgh1* mutant was completely blocked at the initial stages. It is possible that the Sgh1 plays a critical role in the processes that is required for proto-perithecium formation. Moreover, the *SGH1* gene is essential for plant infection, since the Δ*sgh1* mutant was almost non-pathogenic in the infection assays with the flowering wheat heads. The reduced growth of the Δ*sgh1* mutant may partially contribute to its loss of pathogenicity. In addition, the defects of the Δ*sgh1* mutant in the DON biosynthesis and the infection cushion formation may also contribute to its non-pathogenicity.

Considering the critical roles of SR proteins in pre-mRNA processing, an RNA-seq analysis was performed. In the vegetative hyphae, a total of 325 differential splicing events were detected in the Δ*sgh1*mutant in comparison to the wild type, indicating that the Sgh1 appears to be involved in regulating pre-mRNA splicing. Compared with the FgPrp4, which is the only kinase among the spliceosome components, the Sgh1 appears to play a minor role in pre-mRNA splicing, since over 7800 intron retention events were detected in the Δ*Fgprp4* mutant [50]. It is also possible that the Sgh1 was involved in pre-mRNA splicing quality control, becausethe leakage of the unspliced pre-mRNAs into the cytoplasm also causes increased intron retention.Consistent with these findings, both the budding yeast Gbp2p and the Hrb1 prevent the export of mRNA to the cytoplasm until the splicing of introns is completed [51]. Intron retention is associated with lower protein levels, due to intron-retaining transcripts being either degraded by a nonsense-mediated mRNA decay (NMD) pathway or not actively translated [52]. Furthermore, the deletion of the*SGH1* also affected the expression level of over 1110 genes in *F. graminearum*, including many genes that are required for vegetative growth, sexual reproduction, and pathogenicity, such as the protein kinase *FGK3* and *FgYAK1*, transcription factor *FgMCM1*, and the ATP citrate lyase gene *ACL2* [26,44,46]. Although the RNA-seq analysis was performed with vegetative hyphae from culture conditions, it would be expected that the RNA splicing efficiency, or the expression level of more virulence genes, could be affected in the Δ*sgh1* mutant under infection conditions. The altered mRNA splicing events and these down- or up-regulated genes in the Δ*sgh1* likely contribute to the pleiotropic defects of the Δ*sgh1* mutant.

The Sgh1-GFP was localized mainly in the nucleus, which is consistent with its likely functions in RNA processing. In *F. graminearum*, the deletion of the RS region strongly impaired the nuclear translocation of the Sgh1^ΔRS^-GFP compared with the Sgh1-GFP control, indicating that the RS domain is required for the nuclear localization of the Sgh1. Accordingly, one putative NLS was predicted in the RS domain of the Sgh1 by NLStradamus software. In agreement with this, in *S. cerevisiae*, the SR domains of Gbp2 and Hrb1 are important for the nuclear reimport that is mediated by the import receptor Mtr10 [53]. In many other SR proteins, the RS domain also hasbeen shown to function as an NLS [54]. Additionally, in *F. graminearum*, the deletion of the RS region of Sgh1 also caused severe defects in plant infection but minor effects on the vegetative growth and sexual reproduction, which may be attributed to the impaired nuclear translocation of the Sgh1. In general, multisite phosphorylation at the RS region is required for the subcellular localization and functions of SR proteins [55]. Within the RS domain of Sgh1, we identified nine putative serine phosphorylation sites. It is likely that the phosphorylation of the RS domain of Sgh1 is required for its nuclear localization. It is also possible that the RS domain of Sgh1 may function via mediating its interaction with other proteins. It has been reported that the RS domains of the SR proteins participate in protein interactions with a number of other RS-domain-containing splicing factors [19]. In *F. graminearum*, the SR proteins FgSrp1 and FgSrp2 interact with each other to form a complex in vivo [24], which may be mediated by their RS domains. Nevertheless, when the RS domain was absent, partial Sgh1-GFP signals were still localized in the nucleus, indicating that the other parts of Sgh1 could also contribute to the nuclear localization of Sgh1.

The typical SR proteins contain one or two RRM domains that can bind to pre-mRNA [56,57]. Interestingly, the Sgh1 has three RRM domains that may have their own RNA-binding specificity and may interact independently with distinct RNA elements in pre-mRNA. The deletion of each RRM domain resulted in different degrees of defects in the vegetative growth, sexual reproduction, and pathogenicity in *F. graminearum*. However, among them, the RRM3 domain deletion caused the most severe defects in the vegetative growth, sexual reproduction, and pathogenicity. In *S. cerevisiae*, the Gbp2 binds preferentially with RNA via the RRM1–RRM2 tandem, while the RRM3 does not interact with RNA, but serves as a protein–protein interaction platform, which is crucial for the association with the THO/TREX complex [58]. The TREX complex is required for the co-transcriptional recruitment of Hrb1/Gbp2 to nascent mRNA [59]. Therefore, these three RRM domains of the Sgh1 may play distinct roles in the RNA-binding and the protein–protein interactions. It will be important to identify and characterize the individual RRM-binding mRNAs or proteins, which could provide more information for us to understand the function of the Sgh1 in *F. graminearum*.

The function of the SR-specific protein kinases in regulating the nuclear targeting of SR proteins is conserved from fission yeast to humans [55]. Our study has also revealed that the unique SRPK Srk1 physically interacts with the Sgh1 in *F. graminearum*. Thus, we have examined whether the deletion of the *SRK1* gene had any effect on the subcellular localization of the Sgh1. Unexpectedly, the subcellular localization of Sgh1-GFP did not alter in the Δ*srk1* mutant, indicating that the Srk1 is dispensable for the subcellular localization of the Sgh1 in *F. graminearum*. In agreement with this, the FgSrp2 also has an RS-rich region, but its subcellular localization was not regulated by the Srk1 or the Prp4 kinase [24]. In *S. cerevisiae*, the phosphorylation and the nuclear localization of Hrb1 are also independent of the SR-specific protein kinase Sky1, although the cellular localization of Npl3 and Gbp2 is regulated by Sky1 phosphorylation [60]. Therefore, other kinases may regulate the nuclear localization of the Sgh1, while the Srk1 may regulate the RNA binding activity of the Sgh1 or its interaction with other proteins in *F. graminearum*. In *S. cerevisiae* and *C. albicans*, the SR-like proteins Npl3 and Slr1 are methylated at the arginine residues of the RGG box, which regulates their nuclear export, while the unmethylated RGG facilitates their nuclear localization [61,62]. However, the methylation of the RGG motif facilitates the nuclear import of some of the RNA-binding proteins [63,64], also suggesting a role for methylation in nuclear import. Although it is not clear whether the Sgh1 is methylated or not, the predominant arginine methyltransferase Amt1 in *F. graminearum* is important for plant infection [30]. In future studies, we will determine the function of the RGG-box with five RGG repeats in the Sgh1, which may regulate the nucleo-cytoplasmic shuttling of the Sgh1 in *F. graminearum*.

## Data Availability

Not applicable.

## Supplementary Materials

The following are available online at https://www.mdpi.com/article/10.3390/jof8101056/s1, Figure S1. Sequence alignment of the Sgh1 and its orthologs from other fungi. The amino acid sequences of the Sgh1 and its orthologs from *F. oxysporum* (Fo), *M. oryzae* (Mo), *A. nidulans* (An), *U. maydis* (Um), *C. albicans* (Ca), and *S. cerevisiae* (Gbp2 and Hrb1) were aligned with Clustal X 2.1. Identical and similar amino acid residues are shaded in black and gray, respectively. The RS, RRM1, RRM2, and RRM3 domains are marked by black overlines and five RGG repeats are indicated by blue overlines. A putative NLS (nuclear localization signal) is denoted by a red box. Figure S2. The identification of Hrb1/Gbp2 ortholog in *F. graminearum*. A. Comparison of the domain organization of *F. graminearum* Sgh1, *S. cerevisiae* Npl3, Hrb1, and Gbp2, and *S. pombe* Srp1 and Srp1. RRM, RNA recognition motif; RS, arginine/serine-rich domain; R-rich, arginine-rich domain. B. The expression levels (transcripts per kilobase million, TPM) of *SGH1* were estimated with RNA-seq data of conidia (Coni), 12-h hyphae (Hyp12h), infected wheat heads at 1, 2, and 3 dpi (Inf1d, Inf2d, and Inf3d), and perithecia collected at 3 and 6 dpf (Sex3d and Sex6d). The error bars indicate standard deviation calculated from two or three biological replicates of RNA-seq data. Figure S3. The *SGH1* gene replacement construct and deletion mutants. A. The *SGH1* locus and gene replacement construct. The *SGH1* and *hph* genes are marked with empty and black arrows, respectively. The 1F, 2R, 3F, and 4R are primers used to amplify the flanking sequences. *Xho* I (X). B. Southern blot analysis with the wild type (PH-1) and the Δ*sgh1* transformants (M6, M14, and M16). All of the DNA samples were digested with *Xho* I. The blots were hybridized with probe A, which was amplified with primers 1F and 2R. Figure S4. The defects of the three Δ*sgh1* mutants in colony morphology, conidiation, and sexual reproduction. A.The wild type (PH-1) and the three Δ*sgh1* mutants (M6, M14, and M16), which were confirmed by Southern blot analysis, were cultured on CM plates for 60 h. B. The conidia of the same set of strains that were harvested from 5-day-old CMC cultures were imaged by DIC microscopy. Bar = 10 μm. C. The conidial concentrations of the same set of strains were measured in 5-day-old CMC cultures. The different letters indicate the statistically significant differences by Duncan’s multiple range test (*p* = 0.05). D. The perithecium formation on carrot agar cultures of the indicated strains was examined at 7 dpf.Figure S5. The subcellular localization of the Sgh1-, Sgh1^ΔRRM1^-, Sgh1^ΔRRM2^-, and Sgh1^ΔRRM3^-GFP fusion proteins. A. The conidia of the Δ*sgh1*/*SGH1*^ΔRRM1^-, Δ*sgh1*/*SGH1*^ΔRRM2^-, and Δ*sgh1*/*SGH1*^ΔRRM3^-GFP transformants were stained with DAPI and examined by differential interference contrast (DIC) and epifluorescence microscopy. Bar = 10 μm. B. The 12 h hyphae of the same set of strains were stained with DAPI and examined witha Zeiss LSM880 confocal microscope. Bar =10 μm. Dataset S1. A list of differential alternative splicing events in the Δ*sgh1* mutant. Dataset S2. The genes differently expressed in the Δ*sgh1* mutant. Table S1. The PCR primers used in this study.
