# Supplementary material for: Sgh1, an SR-like Protein, Is Involved in Fungal Development, Plant Infection, and Pre-mRNA Processing in Fusarium graminearum
Source: J Fungi (Basel). 2022 Oct 8;8(10):1056. doi: 10.3390/jof8101056 (PMC9605648; doi:10.3390/jof8101056)
Supplement: Supplementary file 1 [file jof-08-01056-s001.zip › Table S1.pdf]

Table S1. PCR primers used in this study

Primers	Sequence (5'-3')
SGH1-1F	GCCTATCCAAGCCATGATCTGAGT
SGH1-2R	TTGACCTCCACTAGCTCCAGCCAAGCCAATGCTGTGCAGAATGAGGGGTA
SGH1-3F	GAATAGAGTAGATGCCGACCGCGGGTTTCAATCAAGGCGAGCGGTTAT
SGH1-4R	CGGGCAAGGGAGATCAGGAAT
SGH1-5F	GCCCAACTGAGCAACCAGAA
SGH1-6R	CCGACCACCGAAACCCATAC
SGH1-7F	GTCACTCGTCGCTCACAT
SGH1-8R	GCCGTTTCTTATTGGCTCATCT
HYG-F	GGCTTGGCTGGAGCTAGTGGAGGTCAA
HYG-R	AACCCGCGGTCGGCATCTACTCTATTC
H852	AACTCACCGCGACGTCTGTC
H850	TTGTCCGTCAGGACATTGTT
H855R:	GCTGATCTGACCAGTTGC
H856F	GTCGATGCGACGCAATCGT
YG-F	GATGTAGGAGGGCGTGGATATGTCCT
HY-R	GTATTGACCGATTCCTTGCGGTCCGAA
SGH1-GFP/F	AGGGAACAAAAGCTGGGTACCCGTCGCTCACATTACCTACCAAC
SGH1-GFP/R	GAACAGCTCCTCGCCCTTGCTCACCATGATCTGATCCTGAGTCAGGC
DRS1R	CGGCTCGCTCTTCGTACTCACGCTGGAGATTGTTAGCTG
DRS2F	CAATCTCCAGCGTGAGTACGAAGAGCGAGCCGTCG
RRM1-DR	GACTGTTATTACTTACCTCTCGGCGGTCCTGCTGG
RRM1-DF	CCAGCAGGACCGCCGAGAGGTAAGTAATAACAGTCGCAAGTCAG
RRM2-DR	TACCGGCGTAGCGGTCCTCACAAACGTTGGCCACATAGATCTG
RRM2-DF	CTATGTGGCCAACGTTTGTGAGGACCGCTACGCCGGT
RRM3-DR	TACTTCACGAAACTCAAATTGATCTCTCCTCTGTCACTGCCG
RRM3-DF	GCAGTGACAGAGGAGAGATCAATTTGAGTTTCGTGAAGTATCTCAACCAAG

SRK1-CU/MF	AAGAACGCGGCCATTACGGCCATGGCCCACTCCCCCTCAT
SRK1-CU/MR	CGACATGGCCGAGGCGGCCAATCTCTTCCTCACCTCCGTCGA
SGH1-NU/F	GATGTTCCAGATTACGCTGGATCCATGGCACCCGCACTTGCA
SGH1-NU/R	ATCGATAAGCTTGATATCGAATTCTTACATGATCTGATCCTGAGTCAGG
SGH1-CYFP/F	CGACTCACTATAGGGCGAATTGGGTACTCAAATTGGTCCTGGACGCTGGTATCTGGT
SGH1-CYFP/R	GTTCGGGATCTTGCAGGCCGGGCGCATGATCTGATCCTGAGTCAGGC
SRK1-NYFP/F	CGACTCACTATAGGGCGAATTGGGTACTCAAATTGGCGTGGCATCCTGTATCACCTCA
SRK1-NYFP/R	GCTCACCATCGTGGCGATGGAGCGTCTCTTCCTCACCTCCGTCGAC
