# Supplementary figures and images for: Sgh1, an SR-like Protein, Is Involved in Fungal Development, Plant Infection, and Pre-mRNA Processing in Fusarium graminearum
Source: J Fungi (Basel). 2022 Oct 8;8(10):1056. doi: 10.3390/jof8101056 (PMC9605648; doi:10.3390/jof8101056)

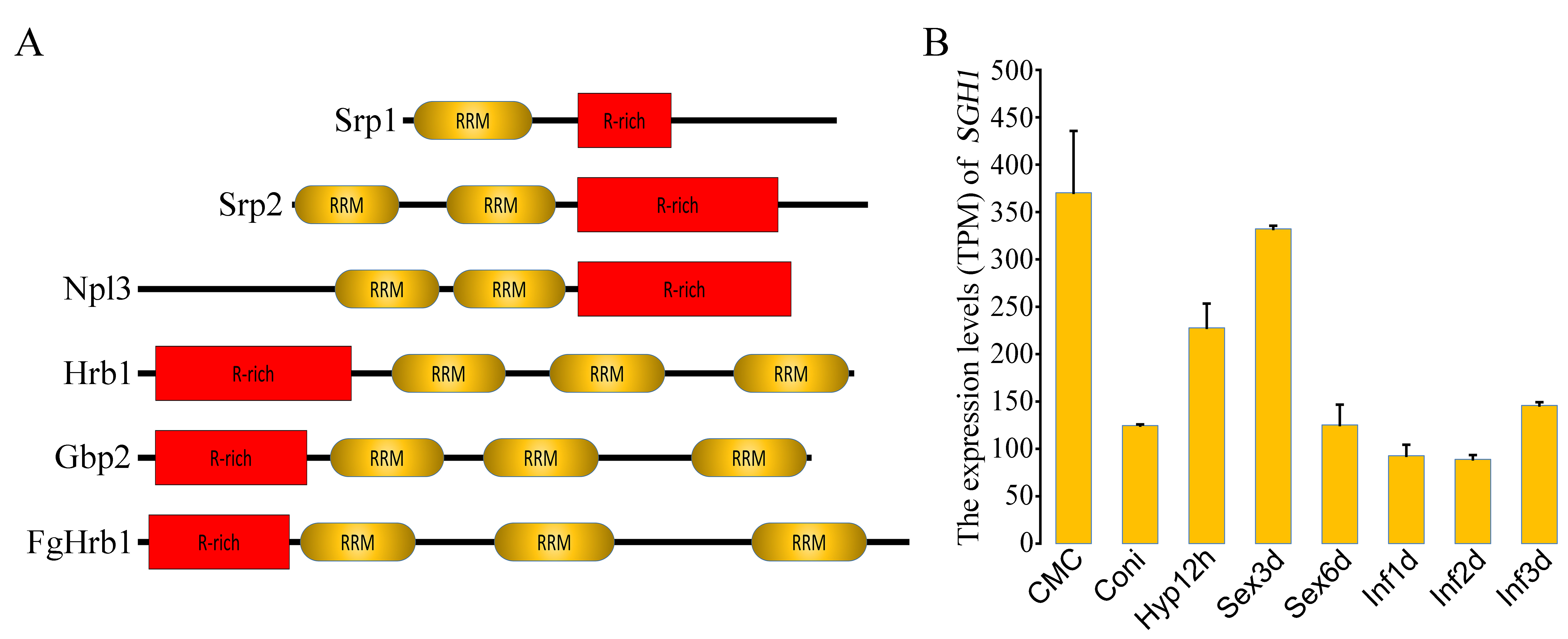

Supplement: Supplementary file 1 [file jof-08-01056-s001.zip › Fig S2 revised.jpg]

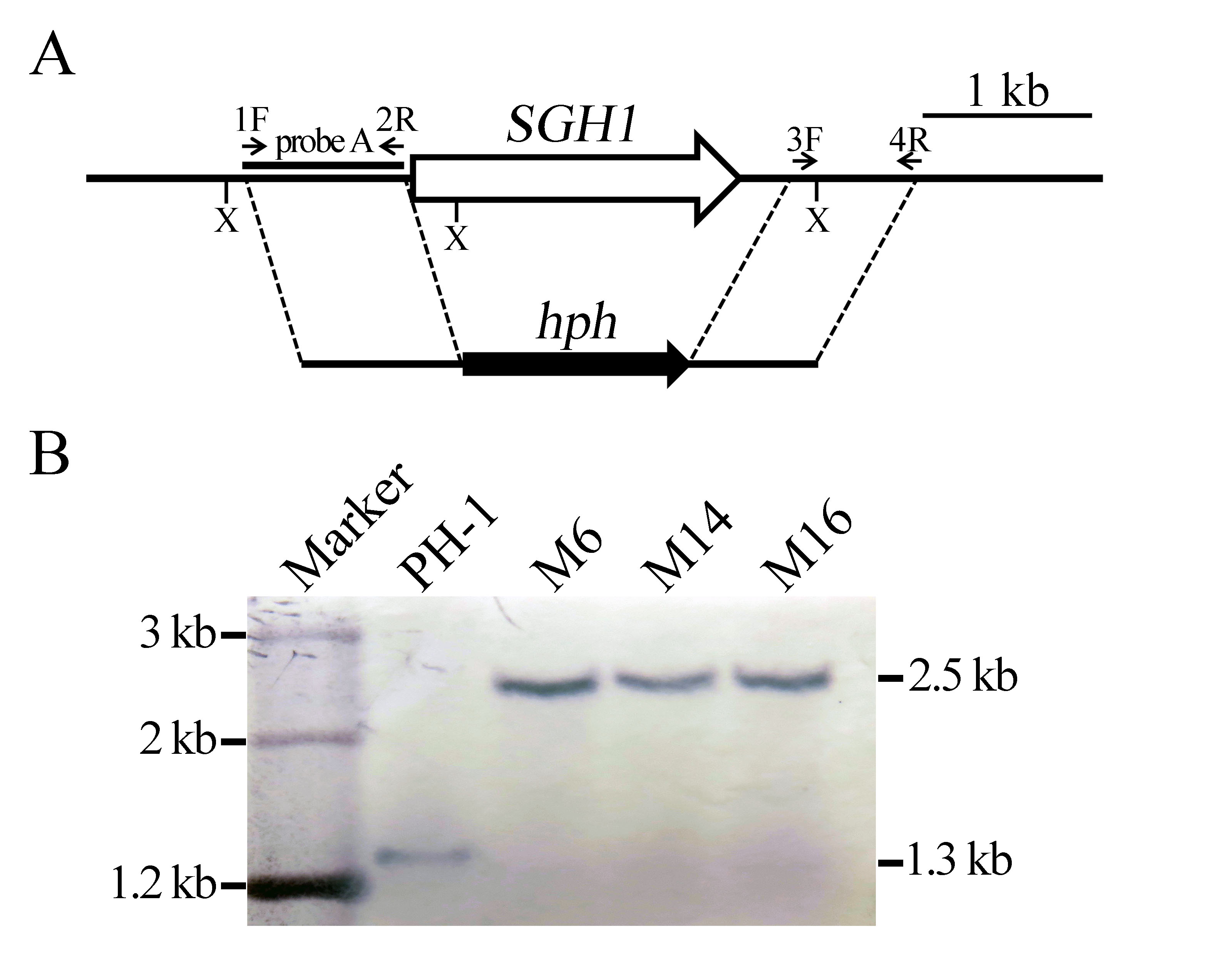

Supplement: Supplementary file 1 [file jof-08-01056-s001.zip › Fig S3.jpg]

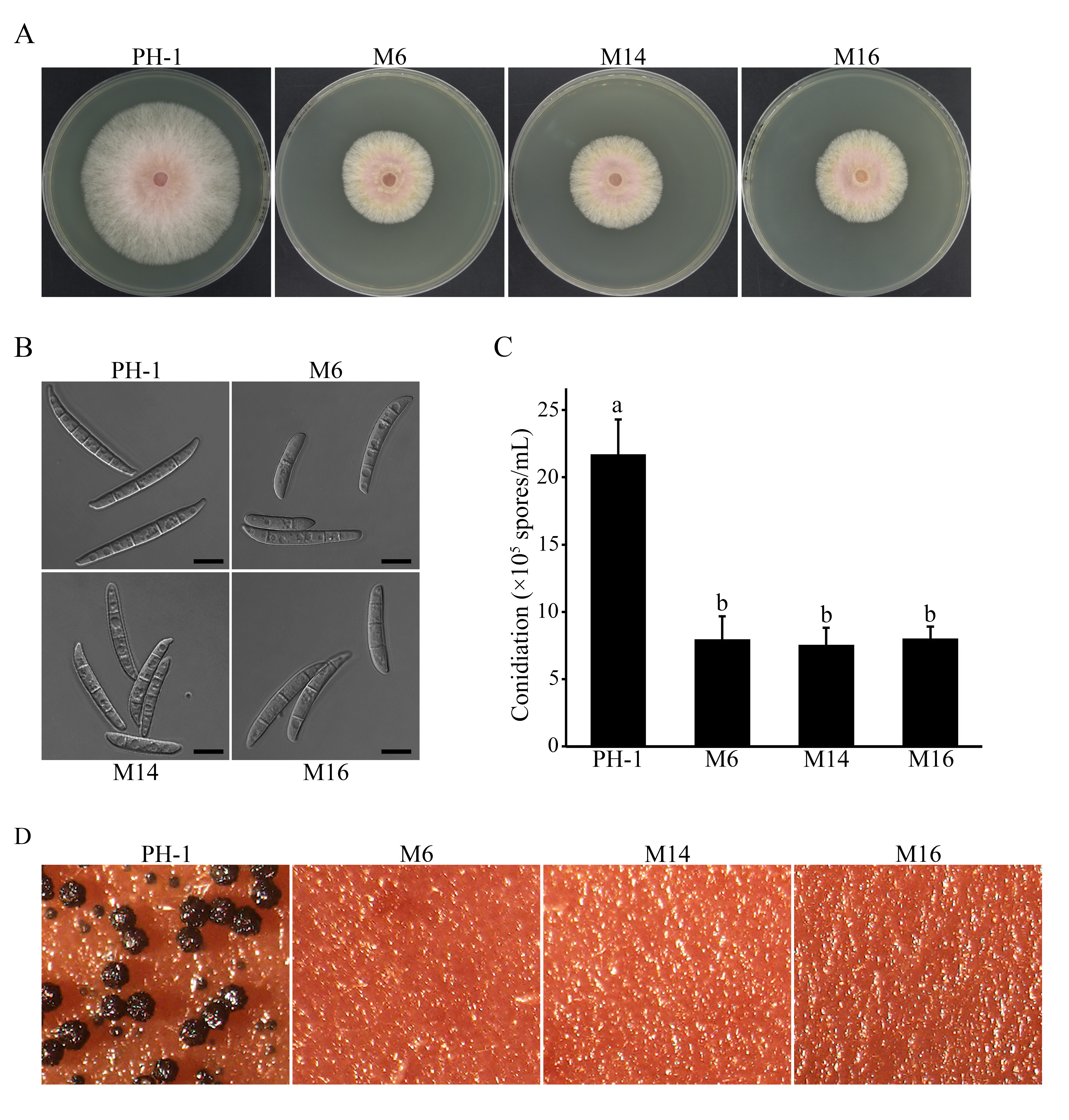

Supplement: Supplementary file 1 [file jof-08-01056-s001.zip › fig S4 new.jpg]

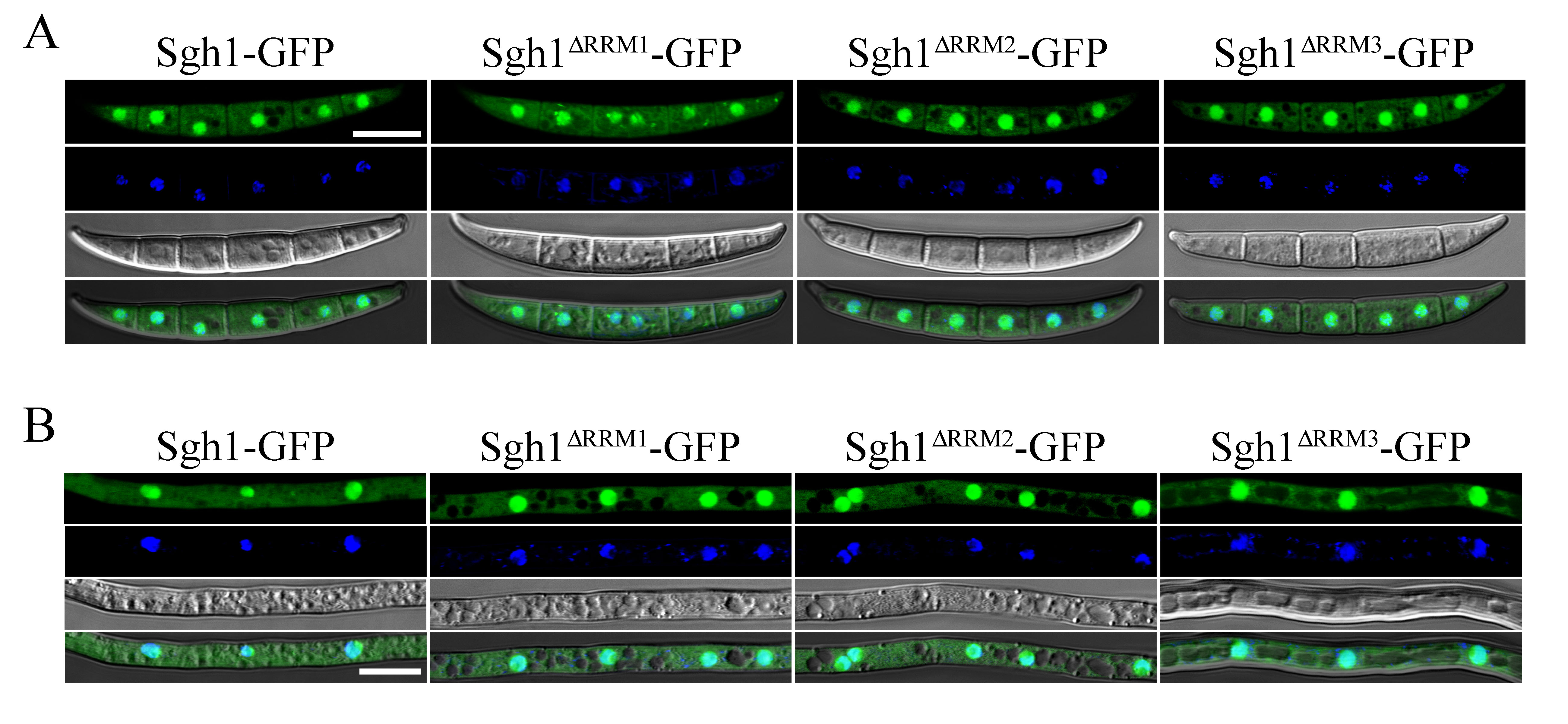

Supplement: Supplementary file 1 [file jof-08-01056-s001.zip › Fig S5.jpg]
