# Supplementary material for: Sgh1, an SR-like Protein, Is Involved in Fungal Development, Plant Infection, and Pre-mRNA Processing in Fusarium graminearum
Source: J Fungi (Basel). 2022 Oct 8;8(10):1056. doi: 10.3390/jof8101056 (PMC9605648; doi:10.3390/jof8101056)
Supplement: Supplementary file 1 [file Fig S1.pdf › Fig S1.pdf]

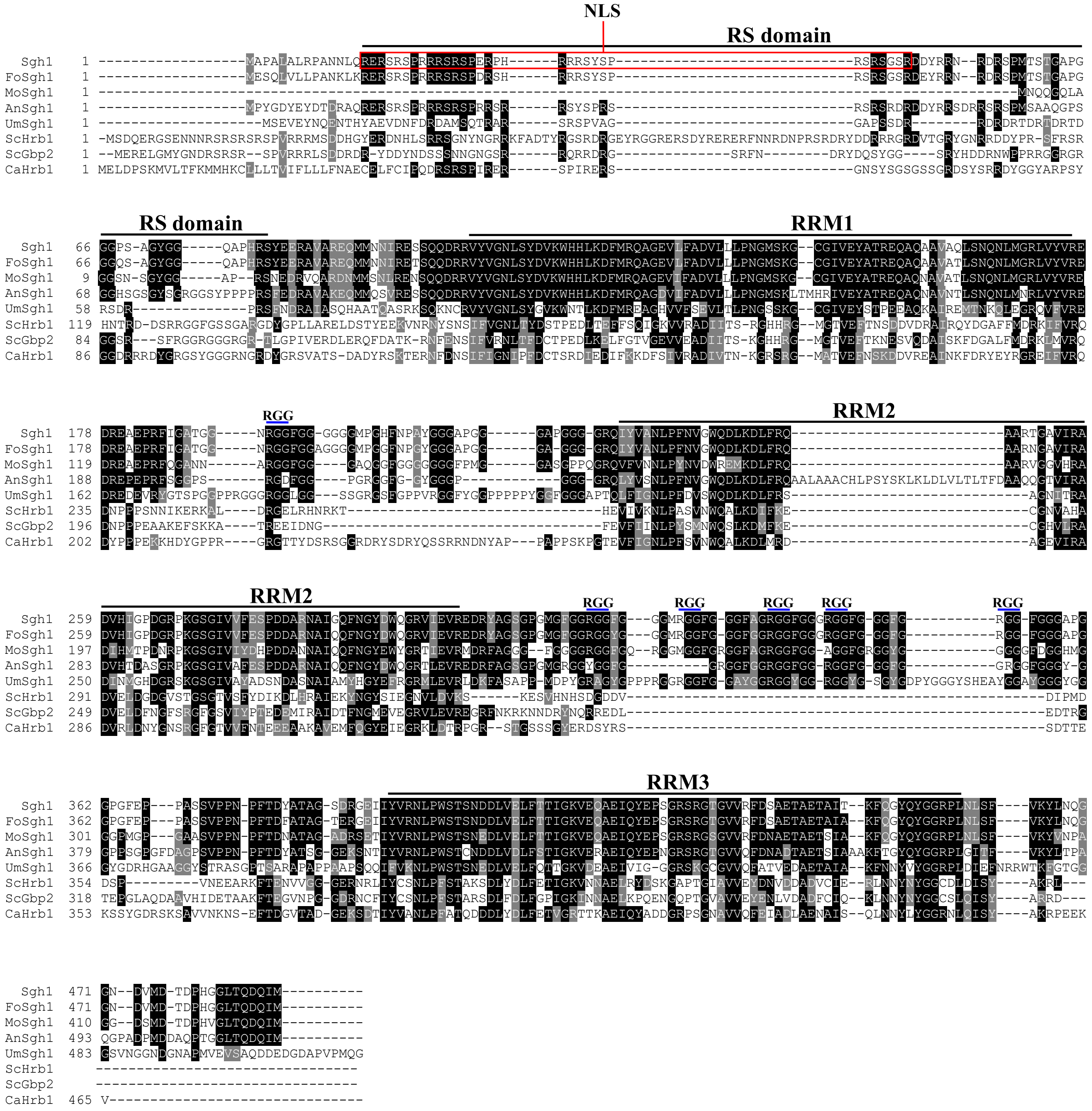
NLS
RS domain
RS domain
RRM1
RGG
RRM2
RRM2
RGG
RGG
RGG
RGG
RGG
RRM3
Sgh1
FoSgh1
MoSgh1
AnSgh1
UmSgh1
ScHrb1
ScGbp2
CaHrb1
